# A Novel Star Like Eight-Arm Polyethylene Glycol-Deferoxamine Conjugate for Iron Overload Therapy

**DOI:** 10.3390/pharmaceutics12040329

**Published:** 2020-04-07

**Authors:** Bohong Yu, Yinxian Yang, Qi Liu, Aiyan Zhan, Yang Yang, Hongzhuo Liu

**Affiliations:** 1 Wuya college of innovation, Shenyang Pharmaceutical University, No.103, Wenhua Road, Shenyang 110016, China; 2 Collage of Pharmacy, Shenyang Pharmaceutical University, No.103, Wenhua Road, Shenyang 110016, China

**Keywords:** 8-arm-polyethylene glycol, deferoxamine, conjugation, chelator, cytotoxicity, half-life

## Abstract

The traditional iron chelator deferoxamine (DFO) has been widely used in the treatment of iron overload disease. However, DFO has congenital disadvantages, including a very short circular time and non-negligible toxicity. Herein, we designed a novel multi-arm conjugate for prolonging DFO duration in vivo and reducing cytotoxicity. The star-like 8-arm-polyethylene glycol (8-arm-PEG) was used as the macromolecular scaffold, and DFO molecules were bound to the terminals of the PEG branches via amide bonds. The conjugates displayed comparable iron binding ability to the free DFO. Furthermore, these macromolecule conjugates could significantly reduce the cytotoxicity of the free DFO, and showed satisfactory iron clearance capability in the iron overloaded macrophage RAW 246.7. The plasma half-life of the 8-arm-PEG-DFO conjugate was about 190 times than that of DFO when applied to an intravenously administered rat model. In conclusion, research indicated that these star-like PEG-based conjugates could be promising candidates as long circulating, less toxic iron chelators.

## 1. Introduction

Chelation therapy has been widely used in the treatment of iron overload, especially when the patients received a regular blood transfusion, such as for thalassemia [1,2]. Blood transfusion adds to the iron burden in patients with high risk of the damage of the liver, endocrine system, and heart. Note that patients with the transfusion-dependent blood disorders who received no chelation therapy had a mean survival time of 12 to 17 years, and death occurred mainly from cardiac failure or arrhythmia due to iron overload [3].

Deferoxamine (DFO), the first approved drug in iron chelation therapy, has a special status in clinical reasearch, due to its long-standing impact [4]. However, the extremely short half-life of DFO (about 5.5 min) means it has to be given intravenously or subcutaneously up to 8–12 h for a single administration, at least 5 days a week [5,6,7,8]. Failure to adhere to a regimen was found to be the major cause of death in the patients with thalassemia [9]. Although the oral iron chelators, such as deferiprone and deferasirox, have been approved in related treatments, they have shown significant toxicities, such as gastrointestinal bleeding, thrombocytopenia, and agranulocytosis—and the latter was reported to be associated the lethal adverse events [10,11,12,13,14]. Therefore, DFO has been licensed as first-line therapy for the treatment of iron overload in many countries. The strategy to improve pharmacokinetics (PK) of DFO was thus regarded as a promising approach to promote therapeutic efficacy and mitigate the side effects.

Many attempts have been conducted by conjugating DFO to a water-soluble polymer to improve its PK properties. For example, the hyper-branched glycerol was used to form conjugates with DFO, which made it possible to chelate iron efficiently and prolong the circulation time of API in the blood [7,15,16]. Specially, the circulation half-life (t_1/2_) of DFO was increased by up to 44 h in mice, a 484-fold increase compared to native DFO. Nevertheless, it has not yet been pursued in clinical study, probably due to the limitations of the excipients and cumbersome synthesis methods. Other DFO macromolecular conjugates, such as polyrotaxane-DFO and dextran-DFO conjugate, also showed efficacies in iron chelation and rather low cell toxicities [17,18]. However, the reported t_1/2_ was prolonged to be a moderate level (~1 h). The PEG has been used in the many products since it was approved by the FDA as a pharmaceutical polymer material [19,20,21]. The PEGylation conjugates provide a solution to increase the stability of drugs and prolong their blood circulation. Tremendous efforts have been devoted to create PEG-based solutions for improving the defect of the DFO. In previous studies, the DFO was conjugated to the copolymer with the PEG side chain or the PEG based three-arm macromolecule, for designing the biocompatible, long-circulating macromolecule iron-chelator [22,23]. These polymer conjugates, however, suffered from the wide molecular range and low loading efficacy of API, making the following translations difficult. Recently, multi-armed PEGs have gained much attention in both academic and industrial fields, due to their superiority in drug-loading efficacies compared to the traditional linear PEG [24,25,26]. Herein, we successfully synthesized and designed an 8-arm-polyethylene glycol-DFO (8-arm-PEG-DFO) conjugate that shows excellent biocompatibility and reasonable residence in circulation, and it thus provided a potential clinical alterative to DFO.

## 2. Materials and Methods

### 2.1. Materials

The 8-arm-PEG Succinimidyl Glutarate (8-arm-PEG-SG) was purchased from Jenkem technology CO., LTD. (Beijing, China). The deferoxamine mesylate salt was acquired from the Sigma-Aldrich CO., LLC (Beijing, China). The mouse ferritin ELISA kit was purchased from the Shanghai Jianglai industrial Limited By Share Ltd. (Shanghai, China). The DMEM culture medium, ammonium iron sulfate hexahydeate, penicillin G, sodium, streptomycin sulfate, and the BCA protein assay kit, were obtained from Dalian Meilun Biological Technology Co., Ltd. (Dalian, China).

### 2.2. The Synthesis of 8-Arm-PEG-DFO Conjugates

The deferoxamine mesylate salt and the 8-arm-PEG-SG with different molecular weights (20 kDa and 40 kDa) were dissolved in anhydrous dimethyl sulfoxide. The DIPEA (Sigma, Aldrich) was added into the solution under magnetic stirring. The reaction was carried out under nitrogen at 25 °C for 24 h. The purification process was carried out by dialyzing against deionized water at 25 °C for another 24 h. The solution was freeze-dried to obtain the final products.

### 2.3. The Characterization of the 8-Arm-PEG-DFO Conjugates

The materials and synthetic products were confirmed by using ^1^H NMR spectroscopy (Bruker AV600, 600 MHz, Karlsruhe, Germany) with DMSO-d6 as the solvents. The purity of the 8-arm-PEG-DFO conjugates were analyzed by reversed phase high performance liquid chromatography. The system composed of the HITACHI 5430 diode array detector (Tokyo, Japan), the HITACHI 5130 column oven, the HITACHI 5210 auto sampler, and the HITACHI 5110 pump. The C_18_ reverse-phase column (Ultimate XB-C18, 150 mm × 4.6 mm, Welch, China) was used for the elution of all the samples. The mobile phase contained 39% acetonitrile, 59% 10 mM phosphate buffer (pH = 3.7), and 2% methanol (v/v) for the 8-arm-PEG_20k_-DFO, and 42% acetonitrile, 56% 10 mM phosphate buffer (pH = 3.7), and 2% methanol (v/v) for the 8-arm-PEG_40k_-DFO. One mM of free DFO and 8-arm-PEG-DFO solutions with the equivalent DFO concentration were used as test samples, and all the samples were eluted at 25 °C.

The ultrafiltration centrifugation was also used to confirm the synthesis of the products. Briefly, DFO, the 8-arm-PEG_20k_-DFO or 8-arm-PEG_40k_-DFO solution was vortexed with 10 mM ammonium iron sulfatehexahydrate for 5 min to form the chelates, followed by ultrafiltration centrifugation at 6000 rpm for 8 min. (MWCO 10 KDa).

### 2.4. Iron Binding Properties of the 8-Arm-PEG-DFO Conjugates

The iron binding ability of the 8-arm-PEG-DFO conjugates were investigated by UV-visible spectrophotometry (UV110211 Tianmei Scientific Instrument Co., Ltd. Shanghai, China). The solution of the DFO, 8-arm-PEG_20k_-DFO, or 8-arm-PEG_40k_-DFO at an equal dose of API was stirred with the same volume of 10 mM ammonium iron sulfate hexahydrate for 30 min, and UV-vis spectrums was conducted for each sample. DFO, 8-arm-PEG_20k_-DFO, and 8-arm-PEG_40k_-DFO without Fe^2+^ incubation were also examined for comparison.

### 2.5. In Vitro Hemolysis Test

Blood was collected from rats and diluted 10-fold with 0.9% normal saline, followed by centrifugation at 2000 rpm for 10 min. The red blood cells (RBC) were washed several times until the supernatant was completely clarified. Then, 0.9% normal saline was added to prepare the 2% RBC dispersion for the further experiment. The 2 mg, 10 mg, and 50 mg 8-arm-PEG-DFO conjugates were added to the 10 mL 2% RBC dispersion to obtain suspensions with different final concentrations (0.2, 1.0, and 5 mg/mL respectively). After vortexing, the dispersions were then incubated at 37 °C for 3 h and centrifuged at 2000 rpm for 10 min. The absorbance of the supernatants was measured at 415 nm. The mixture of RBC dispersion with distilled water was used as a positive control (100% hemolysis), whereas the mixture of RBC dispersion with normal saline was used as a negative control (0% hemolysis). The hemolysis (%) was calculated by the following equation:

$$\mathrm{Hemolysis}(\%)=\frac{OD_{sample}-OD_{negative\;control}}{OD_{positive\;control}-OD_{negative\;control}}\times 100\%$$

### 2.6. In Vitro Metabolism Studies

The metabolism studies were carried out as per the method described in a previous report [27]. First, the blood was centrifuged at 10,000 rpm for 5 min at 4 °C to separate the plasma. The Krebs-Ringer bicarbonate (KRB) buffer was prepared with the following ingredients: 2.5 mM CaCl_2_·2H_2_O, 2.4 mM MgSO_4_·7H_2_O, 1.2 mM KH_2_PO_4_, 4.8 mM KCl, 119.0 mM NaCl, 32.5 mM NaHCO_3_, and 5.6 mM D-glucose. The 8-arm-PEG_20k_-DFO and 8-arm-PEG_40k_-DFO with the equivalent amount of DFO were dissolved in the mixed solution of 10 mL KRB buffer and 2 mL plasma. The obtained solution was then vortexed and allowed to be incubated at 37 °C after shaking. The incubated solution was withdrawn at pre-determined time points, followed by deproteinized procedure. The samples were incubated with Fe^2+^ for a period of time to form the iron chelates, and the chelates were analyzed by UV-vis spectroscopy at a wavelength of 430 nm.

### 2.7. Cell Culture

The Mouse monocyte macrophages RAW 246.7 obtained from Dingguo Changsheng Biotechnology co., LTD (Beijing, China) were used for the cell experiments. The macrophages were cultured in a DMEM medium supplemented with 10% FBS at 37 °C in the atmosphere of 5% CO_2_.

### 2.8. Cell Viability Analysis

The cell viability of the 8-arm-PEG_20k_-DFO, 8-arm-PEG_40k_-DFO, and free DFO was investigated by the MTT assay. Briefly, the RAW 246.7 macrophages were seeded in 96-well culture plates at a density of 3 × 10^3^ cells per well and incubated for 12 h. After adhering to the wall, the 8-arm-PEG-DFO conjugate and DFO solutions at the desired concentrations were prepared and replaced the culture medium. After incubation for another 48 h, the cells were rinsed with a PBS buffer three times to wash off the 8-arm-PEG-DFO conjugates or DFO. Twenty μL of MTT solution was then added into each well and continued to incubate for 4 h in the dark. Finally, the solution in each well was removed and replaced with 200 μL DMSO, and the absorbance of each sample was measured at 570 nm with a microplate reader. The cells without the treatment of the drug were used as a control. The cell viability (%) was evaluated by the equation:

$$\mathrm{Cell}\;\mathrm{viability}\;(\%)=\frac{OD_{sample}-OD_{blank}}{OD_{control}-OD_{blank}}\times 100\%$$

### 2.9. Iron Chelation Studies in Iron-Overload Macrophages

The RAW 246.7 macrophages were seeded in 6-well plates at a density of 5 × 10^4^ cells per well and cultured for 24 h. According to the previous studies, to induce cell iron overload without affecting their viability, the macrophages were cultured in DMEM, which contained a 100 μM FAC complete medium for 24 h after attachment [18,28]. Subsequently, the previous medium was discarded and the macrophages were washed several times with PBS to remove excess iron. Then, the macrophages were treated with 8-arm-PEG_20k_-DFO, 8-arm-PEG_40k_-DFO, and DFO at 10 μM or 50 μM (the DFO conjugates and DFO were dissolved in the DMEM complete medium) for 48 h. Finally, the macrophages were lysed, and the total protein concentration was measured with the BCA protein assay kit, and the cellular ferritin concentration was measured with a mouse ferritin ELISA kit. The results were expressed by the amount of ferritin per the total protein.

### 2.10. Pharmacokinetics Study

#### 2.10.1. Animal Studies

Pharmacokinetics study was performed in male SD rats from the Experimental Animal Center of Shenyang Pharmaceutical University (Shenyang, China, 220–250 g). The SD rats were fasted for 12 h before the experiments and divided into three groups randomly (six rats each group). The 8-arm-PEG_20k_-DFO, 8-arm-PEG_40k_-DFO, and free DFO with equivalent to 50 mg/kg DFO were intravenously administered to the rats via the tail vein. The blood samples were collected at 0.08, 0.5, 1, 2, 4, 6, 12, and 24 h post-dosing into heparinized tubes and immediately centrifuged at 13,000 rpm for 5 min to obtain the plasma. The animal experimental protocols were approved by the Shenyang Pharmaceutical University Institutional Animal Care and Use Committee (SYPU-IACUC-C2019-8-14-201).

#### 2.10.2. Sample Preparation and Chromatography Analysis

The plasma samples (100 μL) were mixed with 10 mM Fe^2+^ aqueous solution (100 μL) and then vortexed for 5 min. Afterwards, the sample were precipitated by ice-cold acetonitrile (400 μL) and centrifuged at 13,000 rpm for 5 min. The supernatants were extracted and dried by nitrogen and re-dissolved by distilled water (100 μL). The concentration of DFO in the samples was analyzed by HPLC, as described above.

### 2.11. Statistical Analysis

All the results were expressed as mean ± SD. The significant difference was determined by t-test and expressed as (* *P* < 0.05), (** *P* < 0.01), (*** *P* < 0.001), and ns (no significance).

## 3. Result and Discussion

### 3.1. The Synthesis and Characterization of 8-Arm-PEG-DFO Conjugates

**Figure 1:**
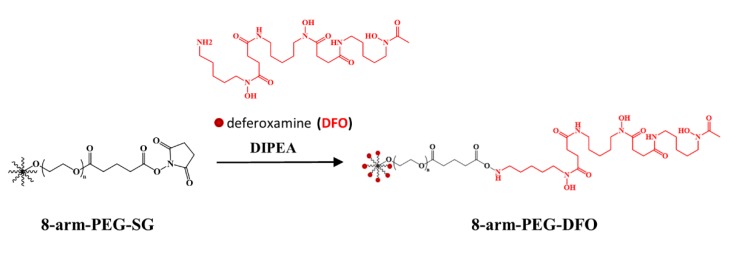
Synthetic route of the 8-arm-polyethylene glycol- deferoxamine (8-arm-PEG-DFO) conjugates.

**Figure 2:**
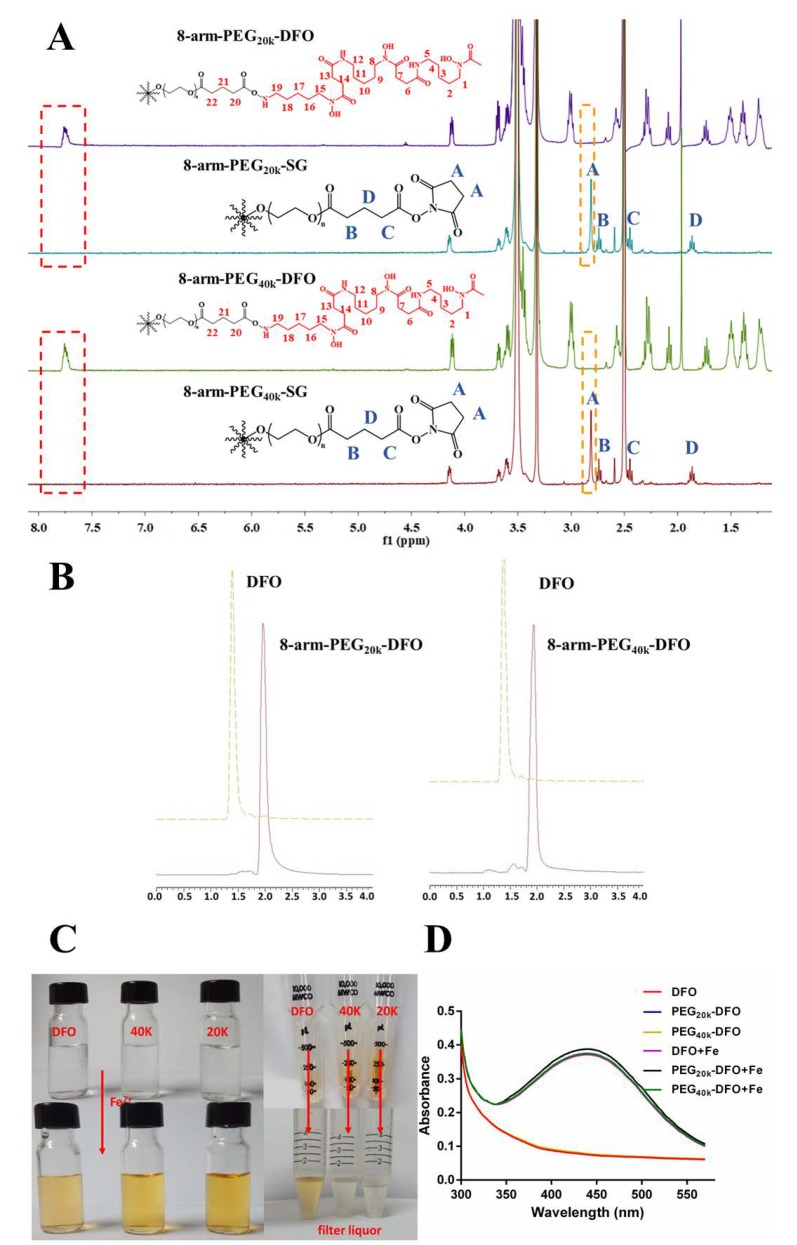
The synthesis and characterization of 8-arm-PEG-DFO conjugates. (**A**) The ^1^H NMR spectrums of the products and the materials. Both of the newly-appeared conjugates peak at 7.75 ppm, which is attributed to the amido bonds and the disappearance of the methylene characteristic peaks at 2.80 ppm on succinimide (peak A), as indicated by the synthesis of the 8-arm-PEG-DFO conjugates. Characteristic peaks of 8-arm-PEG-DFO: (3.4 ppm: H-5, H-12, and H-19; 3.0 ppm: H-1, H-8 and H-15; 2.6 ppm: H-6, H-13; 2.3 ppm: H-7, H-14, H-22; 2.1 ppm: H-20; 1.9 ppm: -CH_3_; 1.7 ppm: H-21; 1.5 ppm: H-4, H-11, and H-18; 1.4 ppm: H-2, H-9, H-16; 1.2 ppm: H-3, H-10, and H-17); (**B**) The HPLC chromatograms for the free DFO and the 8-arm-PEG-DFO conjugates; (**C**) The optical images of DFO, 8-arm-PEG_20k_-DFO, and 8-arm-PEG_40k_-DFO solutions, and the ultrafiltration centrifugation of the DFO-Fe^2+^ and 8-arm-PEG-DFO conjugates-Fe^2+^ chelates; (**D**) UV-visible spectra of aqueous solution of DFO, 8-arm-PEG_20k_-DFO, and 8-arm-PEG_40k_-DFO with Fe (II). Spectra of 8-arm-PEG-DFO conjugates and DFO without Fe (II) were also given.

The 8-arm-PEG-DFO conjugates were synthesized by coupling DFO to an 8-arm-PEG with a mean molecular weight of 20 kDa or 40 kDa, respectively (Figure 1). ^1^HNMR was used to ensure the synthesis of the 8-arm-PEG-DFO conjugates (Figure 2A). In the spectrums of conjugates, the newly-appeared conjugate peak at 7.75 ppm was assigned to the amido bonds, indicating the conjugation of DFO to the 8-arm-PEG macromolecules. Meanwhile, the disappeared characteristic peaks of succinimide at 2.80 ppm further confirmed the successful conjugation. The modification degree of 8-arm-PEG with DFO was nearly ~100%, as determined by the comparison of the integrations of the methylene groups (16H(2.1 ppm: H-20), 16H(1.7 ppm: H-21)) to the integrations of the methylene groups on the DFO (48H(1.5 ppm: H-4, H-11 and H-18), 48H(1.4 ppm: H-2, H-9, H-16), 48H(1.2 ppm: H-3, H-10 and H-17)). The purity of synthesized conjugates was over 94%, as calculated by HPLC (Figure 2B).

The ultrafiltration centrifugation was also used to ensure the conjugation of DFO to the 8-arm-PEG. The solution of free DFO, 8-arm-PEG_20k_-DFO, or 8-arm-PEG_40k_-DFO with the equivalent amount of DFO was mixed with the same volume of Fe^2+^ solution to form chelates, following the ultrafiltration centrifugation. Figure 2C shows the appearance of solution before and after ultrafiltration centrifugation. It was clearly observed that the conjugates were intercepted by the ultrafiltration centrifuge tube, which led the filtered liquid to be colorless. On the other hand, the chelate between free DFO and Fe^2+^, due to their low molecular weight, could penetrate the ultrafiltration centrifuge tube, which made the filtered liquid yellowish.

The capabilities of conjugates to bind Fe^2+^ were characterized by UV-Vis spectroscopy. As shown in Figure 2D, DFO and 8-arm-PEG-DFO conjugates without Fe^2+^ incubation showed no absorption peak around 430 nm [18,29], while the chelates of both DFO-Fe^2+^ and 8-arm-PEG-DFO conjugates-Fe^2+^ displayed a similar absorbance spectrum. It therefore indicated that the iron binding capacity of API was not compromised by the conjugation.

### 3.2. In Vitro Hemolysis Test

**Figure 3:**
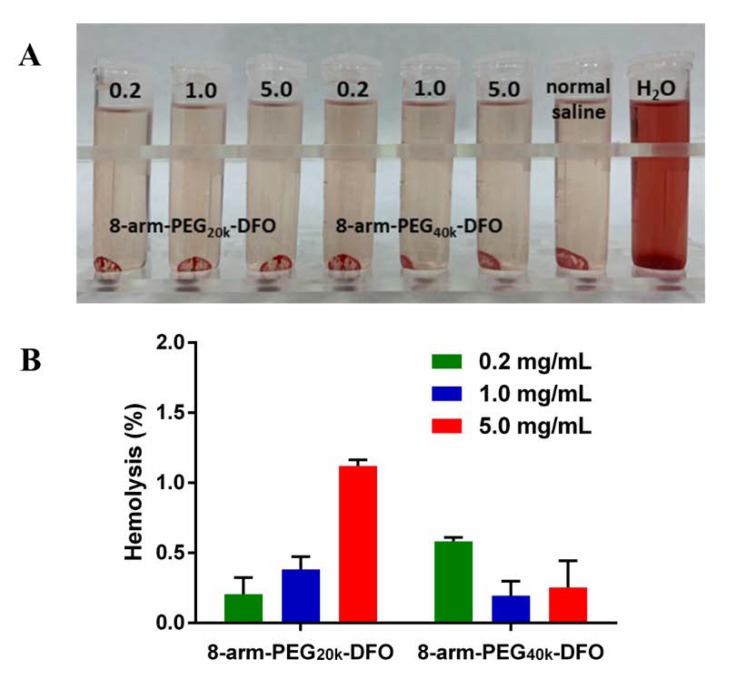
The hemolysis test of the 8-arm-PEG-DFO conjugates. (**A**) The visual appearance of the 8-arm-PEG-DFO conjugates with different contents in red blood cell (RBC) suspension for hemolysis test; (**B**) The hemolysis (%) of the 8-arm-PEG-DFO conjugates with different contents in RBC suspension, even when the concentration reached 5 mg/mL, the conjugates did not show significant hemolysis.

The biocompatibility for the 8-arm-PEG-DFO conjugates were investigated by hemolysis test (Figure 3). The hemolysis character was evaluated by incubating the RBCs dispersion with 8-arm-PEG_20k_-DFO or 8-arm-PEG_40k_-DFO. The photos of hemolysis attributed to the conjugates with different DFO contents were shown in Figure 3A. It was observed that the hemolysis phenomenon of all the conjugates was similar to the normal saline, which indicated the 8-arm-PEG-DFO conjugates did not damage the RBC membrane. Subsequently, accurate hemolysis percentages (%) were calculated, and the result is shown in Figure 3B. Even when the content of 8-arm-PEG_20k_-DFO and 8-arm-PEG_40k_-DFO was added to the concentration of 5mg/mL (equal to 1.6 mM or 0.8 mM of DFO), the hemolysis percentage of the 8-arm-PEG-DFO conjugate was still below 1.2%. The quite low hemolysis activity indicated that the macromolecular conjugates had excellent biocompatibility for intravenous administration [30,31].

### 3.3. In Vitro Metabolism Test

**Figure 4:**
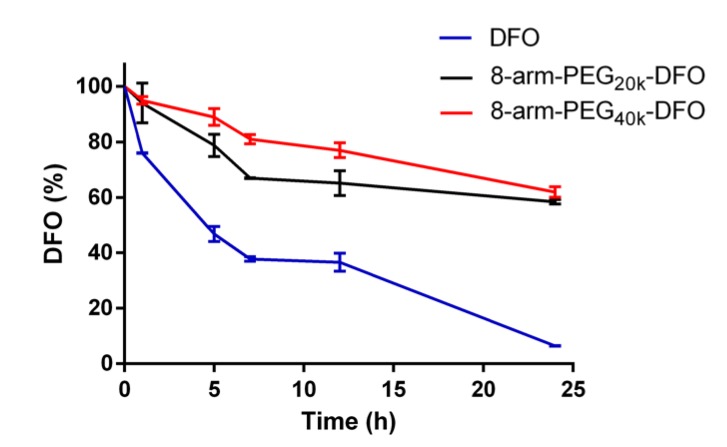
The stability of free DFO, 8-arm-PEG_20k_-DFO, and 8-arm-PEG_40k_-DFO conjugates in plasma.

Due to the existence of the metabolic enzyme, DFO could be rapidly degraded in the plasma and lose the capability of chelating iron [27]. An in vitro plasma metabolism test was then carried out to evaluate the stability of the resultant conjugates. Results of DFO content retained in the conjugates after incubation with plasma are shown in Figure 4. After 24 h incubation, the remaining DFO contents (%) of the free DFO, 8-arm-PEG_20k_-DFO, and 8-arm-PEG_40k_-DFO were 6.4±0.1%, 58.5 ± 0.8%, and 62.0 ± 1.9%, respectively. It demonstrated that the PEG-DFO complexes could significantly delay the drug metabolism and improve the plasma stability of DFO. The 8-arm-PEG is a kind of hydrophilic polymer with many hydroxyl and carboxylic groups along the star-like arms, which inhibits the adsorption of proteins such as enzymes, thereby reducing the degradation of 8-arm-PEG conjugated DFO [27]. In addition, the remaining DFO content of the 8-arm-PEG_40k_-DFO was always higher than the content of the 8-arm-PEG_20k_-DFO after 5 h of incubation, which indicated that the higher molecular weight conjugate had more tendencies to slow the degradation of DFO. This phenomenon was consistent with previous study that found conjugates with larger sizes of PEG exhibited slower clearance rates [32,33].

### 3.4. In Vitro Cytotoxicity Test

**Figure 5:**
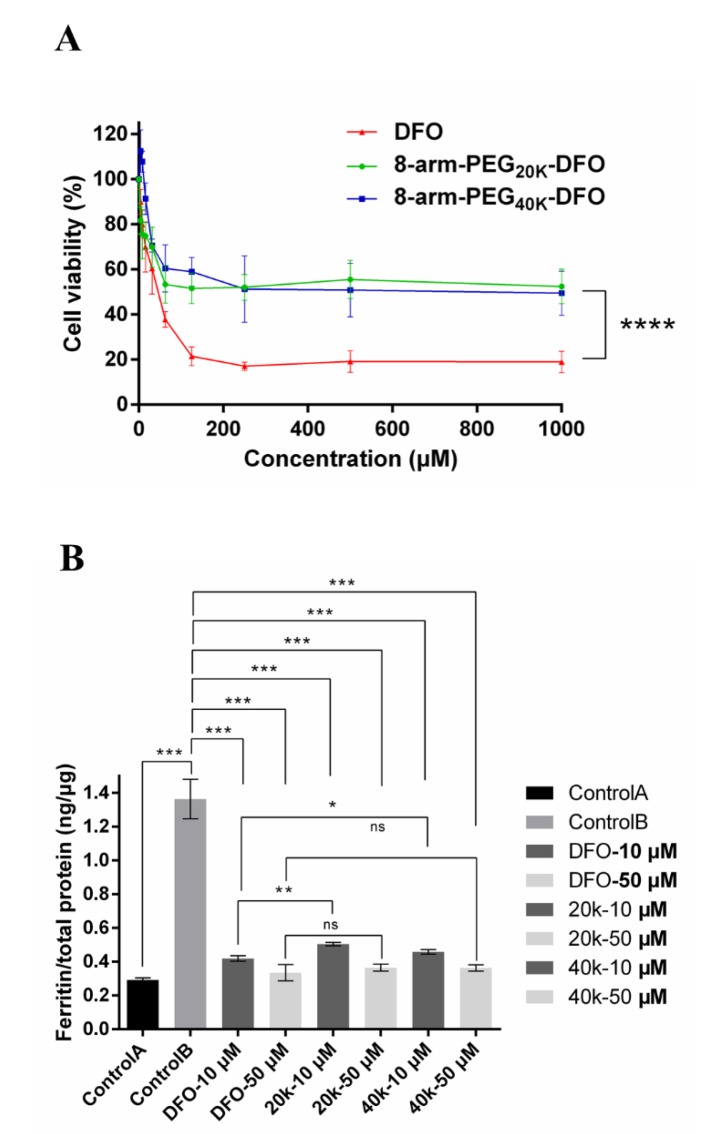
(**A**). In vitro cytotoxicity of DFO, 8-arm-PEG_20k_-DFO, and 8-arm-PEG_40k_-DFO upon the RAW 246.7 macrophage cells; (**B**). The ferritin reduction assay to monitor iron chelation efficacy of DFO, 8-arm-PEG_20K_-DFO, and 8-arm-PEG_40K_-DFO in iron-overloaded RAW 246.7 macrophage cells; cells were treated with DFO or equivalent 8-arm-PEG-DFO conjugates at 10 or 50 μM for 48 h. Cellular ferritin level was measured by the mouse ferritin ELISA assay. Results are normalized to total protein (ng/μg). * *P* < 0.05, ** *P* < 0.01, *** *P* < 0.001, **** *P* < 0.0001.

Previous studies indicated the toxicity of DFO [34,35]. Here, cell viability was evaluated to investigate whether the DFO-PEG conjugates could reduce the related toxicity of API. The solutions of DFO, the 8-arm-PEG_20k_-DFO, or the 8-arm-PEG_40k_-DFO with different concentrations (3.9 to 1000 μM) were incubated with the RAW 246.7 macrophage cells for 48 h, and the result of cytotoxicity is shown in Figure 5A. There was no significant toxicity in the low content range of DFO (below 31.25 μM) in all investigating groups. While when the equivalent DFO concentration was over 62.5 μM, the macrophages incubated with the 8-arm-PEG-DFO conjugates distinctly showed higher cell viability than that of the free DFO. This phenomenon manifested in the 8-arm-PEG macromolecular conjugates could effectively reduce the cytotoxicity of DFO. Notably, there was no significant difference in cytotoxicity between the 8-arm-PEG_20k_-DFO and the 8-arm-PEG_40k_-DFO in the whole concentration range.

### 3.5. Iron Chelation Efficacy Studies in Iron-Overload Macrophages

To further investigate the iron chelation efficacy of the 8-arm-PEG-DFO conjugates, ferric ammonium citrate (FAC) was used to induce the iron-overloaded RAW 246.7 macrophages. Figure 5B demonstrated that the amount of cellular ferritin expression significantly increased after incubation with 100 μM FAC for 48 h (from 0.292 ± 0.012 to 1.364 ± 0.117 ng/μg of total protein), indicating the successful induction of the iron-overloaded RAW 246.7 macrophages (negative control A and positive control B). The iron-overloaded macrophages were then treated with DFO, equivalent 8-arm-PEG_20k_-DFO, or 8-arm-PEG_40k_-DFO to evaluate the chelating ability of the conjugates. Ten μM DFO reduced the cellular ferritin level from 1.364 to 0.419 ng/μg (69.2% decrease), while 50 μM DFO resulted in 0.334 ng/μg of ferrintin in total protein (75.5% decrease). Treatment with 10 μM equivalent 8-arm-PEG_20k_-DFO decreased the cellular ferritin expression level to 0.504 ng/μg (63.1% decrease), and to 0.364 ng/μg total protein for 50 μM (73.3% decrease). In the case of the 8-arm-PEG_40k_-DFO, the cellular ferritin expression level was reduced to 0.458 ng/μg total protein (66.4% decrease) for 10 μM and 0.363 ng /μg total protein for 50 μM (73.4% decrease), respectively.

Free DFO, 8-arm-PEG_20k_-DFO, and 8-arm-PEG_40k_-DFO at both 10 μM and 50 μM equivalent DFO concentrations could significantly decrease the cellular ferritin level in the macrophages, which indicates the conjugated DFO has a strong iron chelation ability. There was no significant difference in the ability of decreasing the cellular ferritin level between 8-arm-PEG-DFO conjugates and the free DFO treatment at the high dose (50 μM). However, the cellular ferritin levels of the 8-arm-PEG_20k_-DFO and 8-arm-PEG_40k_-DFO groups at 10 μM were a little higher than that of the free DFO group (*P* < 0.05), suggesting the 8-arm-PEG-DFO conjugates had a slightly lower iron chelation ability, compared to free DFO at the low dose. This could be due to the large molecular weight of the 8-arm-PEG-DFO, making it more difficult to migrate and achieve ion chelation [27].

### 3.6. Pharmacokinetics Study

**Figure 6:**
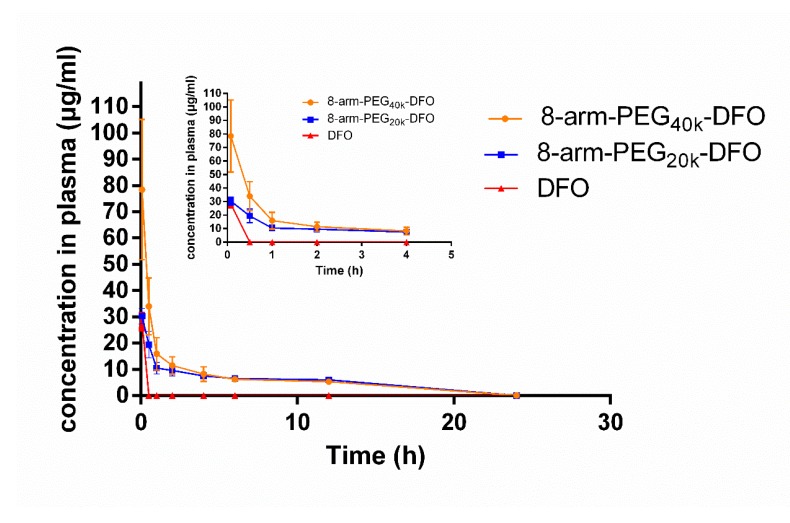
The plasma concentration–time curves of DFO, 8-arm-PEG_20k_-DFO, and 8-arm-PEG_40k_-DFO after intravenous administration in rats. All the concentrations in the curves were normalized to the DFO concentration.

To determine the PK properties of free DFO and the conjugates, the concentrations of DFO in plasma were determined after intravenous injection of free DFO, 8-arm-PEG_20k_-DFO, or 8-arm-PEG_40k_-DFO in rats. The time-dependent plasma concentration curves are shown in Figure 6, and the main parameters were calculated by the conventional two-compartment PK model (Table 1). It was clearly demonstrated that free DFO had a rapid clearance in the blood stream and became undetectable in the plasma at 30 min, which was consistent with the previous reports (a very short circulation half-life (t_1/2_)—about 5.5 min in mice) [7,8]. As expected, the conjugates significantly delayed the plasma clearance time of the DFO. The terminal t_1/2_ of 8-arm-PEG_20k_-DFO and 8-arm-PEG_40k_-DFO were 17.58 ± 8.11 h and 10.13 ± 3.31 h, respectively, which were ~190-fold and ~110-fold of that of the DFO respectively. Specially, the volumes of distribution of the conjugates were 0.5 ~ 0.7 L/kg, indicating extracellular distribution. While DFO possessed larger *V*_d_ (almost total body water in rats and over 1.35 L/kg in human), the result suggested free drugs tend to distribute throughout the body and might interact with intracellular components, thus leading to cellular toxicity [36,37,38]. As compared with the DFO group, the 8-arm-PEG_20k_-DFO group showed comparable *C*_max_, whereas the 8-arm-PEG_40k_-DFOgroup showed a 2.0-fold increase in *C*_max_. It suggested that conjugation with 8-arm-PEG_40k_ might hold a toxic risk of a high dose in the plasma, despite of the prolonged t_1/2_ ability.

## 4. Conclusions

In this study, we designed a novel star-like 8-arm-PEG-DFO conjugate. The synthetic conjugates had the eminent capability of iron binding and the excellent blood biocompatibility. In vitro cell experiments on RAW 246.7 macrophages showed that the conjugates could significantly reduce the cytotoxicity of DFO, and had satisfactory iron clearance ability in iron-overloaded conditions. The in vitro plasma metabolism test and the in vivo pharmacokinetic study both indicated that the 8-arm-PEG-DFO conjugates could significantly prolong the in vivo circulation time of DFO. In summary, these 8-arm-PEG-DFO conjugates provided potential in the treatment of iron overload.

## Figures and Tables

**Table 1:** Pharmacokinetic parameters of 8-arm-PEG_20k_-DFO and 8-arm-PEG_40k_-DFO. All the parameters in Table 1 were equivalent to DFO (eq. DFO).

Conjugate	8-Arm-PEG_20k_-DFO	8-Arm-PEG_40k_-DFO
Eq. DFO AUC_(0-t)_ (mg/L*h)	102.07 ± 10.72	122.81 ± 28.22
Eq. DFO C_max_ (mg/L)	19.39 ± 5.03	33.93 ± 10.80
Eq. DFO t_1/2α_(h)	0.18 ± 0.062	0.26 ± 0.14
Eg. DFO t_1/2β_(h)	17.58 ± 8.11	10.13 ± 3.31
Eq. DFO V (L/kg)	0.73 ± 0.60	0.50 ± 0.36
Eq. DFO CL (L/h/kg)	0.21 ± 0.06	0.27 ± 0.04
